# Falsely increased bispectral index values by convective air warming system during kidney transplantation

**DOI:** 10.12669/pjms.323.9858

**Published:** 2016

**Authors:** Se Hun Kim, Byeong-Cheol Lee, Yong Han Kim

**Affiliations:** 1 Dr. Se Hun Kim, MD. Department of Anesthesiology and Pain Medicine, Haeundae Paik Hospital, Inje University, Busan, South Korea; 2 Dr. Byeong-Cheol Lee, MD. Department of Anesthesiology and Pain Medicine, Haeundae Paik Hospital, Inje University, Busan, South Korea; 3 Prof. Yong Han Kim, MD. Department of Anesthesiology and Pain Medicine, Haeundae Paik Hospital, Inje University, Busan, South Korea

**Keywords:** Artifact, Air warmer, Bispectral index, Depth of anesthesia

## Abstract

Bispectral index (BIS) is a reliable parameter for measuring depth of hypnotic level during anesthesia. Convective air warming system is an effective equipment to maintain normothermia during operation. We report falsely elevated BIS value due to convective air warming system while undergoing kidney transplantation.

## INTRODUCTION

Bispectral index (BIS) has been used to measure sedative-hypnotic depth during anesthesia. However, BIS value may be influenced by several agents or movement to lead misinterpretation.^1^ Anesthesiologist makes an effort to maintain normal body temperature. Convective air warming system (WarmTouch™, Covidien, USA) is a useful device to prevent hypothermia. WarmTouch™ can produce high value of BIS inaccurately. We report a case of inexact reading with BIS influenced by WarmTouch™ during kidney transplantation.

## CASE REPORT

A 38-year-old female patient (height: 153cm, weight: 55kg) was scheduled for kidney transplantation from cadaveric donor. She had been diagnosed with chronic kidney disease about two years ago and on hemodialysis for one year.

**Fig.1:**
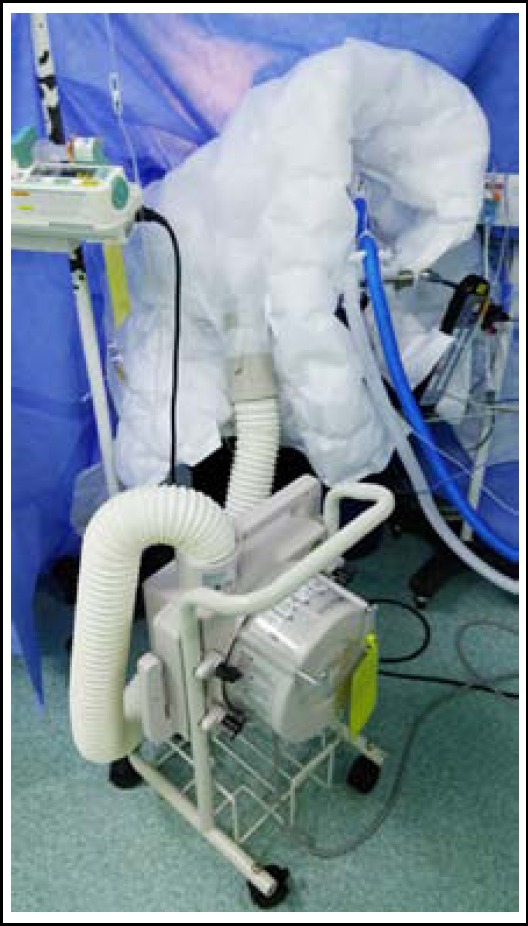
BIS was surrounded by WarmTouch ™ and blanket.

Anesthesia was induced with propofol 100mg and atracurium 25mg with equipment of monitoring including BIS, invasive arterial pressure, pulse oximeter and electrocardiogram. After tracheal intubation, the anesthesia was maintained by 6% desflurane, 0.1μg/kg·min remifentanil and intermittent atracurium. Triple lumen venous catheter was placed in the right internal jugular vein with fluid infusion and central venous pressure monitor. Esophageal probe was inserted to record body temperature. Warming blanket with convective air warming system covered chest, both arms and head to keep body temperature normal (Fig.1). BIS value maintained from 35 to 50 and electromyography (EMG) was almost fixed at 27 during one and half hour of operation. BIS Signal Quality Index (BIS-SQI) and Suppression Ratio (SR) was 100 and 0, respectively. Blood pressure was 131 over 72 mmHg, and heart rate was 69 beats per minute. WarmTouch™ was operated for body heating because esophageal temperature dropped from 36.3 to 36.0ºC. BIS and EMG increased to 74 and 37, respectively. BIS-SQI and SR was 100 and 0, unchanged. Hemodynamics was steady state. The anesthesiologist added up desflurane to 8% concentration and inject atracurium 5mg. However, BIS was over 70 and EMG was 37. After turning off WarmTouch™, BIS and EMG returned to 45 and 27. BIS and EMG was raised again to same numerical value after switch on WarmTouch™. Heated and humidified breathing circuit (Heated Circuit Kit^®^, Ace Medical, South Korea) was applied for normothermia without activation of WarmTouch™. Esophageal temperature was maintained at 36.0 ºC to recovery of anesthesia. Total operation time was four hours and the patient did not recall any intraoperative event. The patient was discharged without any symptom or complication on postoperative 22 days.

## DISCUSSION

BIS monitor is a helpful device for adequate depth of anesthesia during operation. The value of BIS is superior to clinical signs for hypnotic effect of anesthesia.^2^ However, several anesthetics, devices and clinical conditions could affect BIS value.^1^

BIS was increased after ketamine bolus (0.4mg/kg) followed by continuous infusion (1mg/kg·h).^3^ Halothane anesthesia reflects higher BIS than sevoflurane in breast surgery, therefore halothane inhalation based on only BIS has a possibility of overdose.^4^ BIS of halothane at 1 MAC is 56.1 whereas isoflurane is 33.2 in children.^5^

Some of the electronic devices could interfere BIS parameter. Forced air warmer (Bair Hugger^®^, Augustine Medical, USA) elevated BIS without hemodynamic change during hepatectomy with isoflurane, sufentanyl, and atracurium.^6^ Falsely increased BIS was also noted during cardiac surgery heated by Bair Hugger^®^ with sevoflurane, fentanyl and rocuronium anesthesia without memory recall.^7^ Endoscopic shaver attributed falsely elevated BIS with sevoflurane, remifentanil and rocuronium during endoscopic shoulder surgery.^8^ Pacemaker-induced artifact appeared after deep hypothermic circulatory arrest with propofol, remifentanil and rocuronium in cardiac surgery.^9^ BIS increased falsely in patients with electromagnetic operating device (VTI surgery).

Perioperative normothermia is very important to prevent following complications: delayed recovery, myocardial ischemia, shivering, hypertension, tachycardia, and prolonged coagulation time. Various methods could be adapted for maintenance of temperature including forced air blanket, water mattress, warm intravenous fluid, and heated airway circuit.^10^ WarmTouch™ is an effective method for preventing complication of hypothermia. The vibration of air warmer could interfere BIS value through high EMG signal. BIS represented higher value than real depth of hypnotic level. The ventilation outlet of WarmTouch™ was set close to BIS as it seems in Fig.1.

In conclusion, BIS can be interpreted incorrectly by convective air warming system, therefore, deserves a special attention.
